# Nanoformulations of curcumin: an emerging paradigm for improved remedial application

**DOI:** 10.18632/oncotarget.19164

**Published:** 2017-07-11

**Authors:** Meeta Gera, Neelesh Sharma, Mrinmoy Ghosh, Do Luong Huynh, Sung Jin Lee, Taesun Min, Taeho Kwon, Dong Kee Jeong

**Affiliations:** ^1^ Department of Animal Biotechnology, Faculty of Biotechnology, Jeju National University, Jeju, Republic of Korea; ^2^ Division of Veterinary Medicine, Faculty of Veterinary Science and Animal Husbandry, Sher-e-Kashmi University of Agricultural Sciences and Technology, R.S. Pura, Jammu, India; ^3^ Department of Animal Biotechnology, College of Animal Bioscience and Technology, Kangwon National University, Gangwon-do, Republic of Korea; ^4^ Laboratory of Animal Genetic Engineering and Stem Cell Biology, Subtropical/Tropical Organism Gene Bank, Jeju National University, Jeju, Republic of Korea

**Keywords:** curcumin, herb, nano, nanoformulation, nanotechnology

## Abstract

Curcumin is a natural polyphenol and essential curcuminoid derived from the rhizome of the medicinal plant *Curcuma longa* (*L*.) is universally acknowledged as “*Wonder drug of life*”. It is a vital consumable and restorative herb, commonly keened for several ailments such as cancer, arthritis, pain, bruises, gastrointestinal quandaries, swelling and much more. Despite its enormous curative potential, the poor aqueous solubility and consequently, minimal systemic bioavailability with rapid degradation are some of the major factors which restrict the utilization of curcumin at medical perspective. However, to improve its clinically relevant parameters, nanoformulation of curcumin is emerging as a novel substitute for their superior therapeutic modality. It enhances its aqueous solubility and targeted delivery to the tissue of interest that prompts to enhance the bioavailability, better drug conveyance, and more expeditious treatment. Subsequent investigations are endeavored to enhance the bio-distribution of native curcumin by modifying with felicitous nano-carriers for encapsulation. In this review, we specifically focus on the recent nanotechnology based implementations applied for overcoming the innate constraints of native curcumin and additionally the associated challenges which restrict its potential therapeutic applications both *in vivo* and *in-vitro* studies, as well as their detailed mechanism of action, have additionally been discussed.

## INTRODUCTION

**Figure 1:**
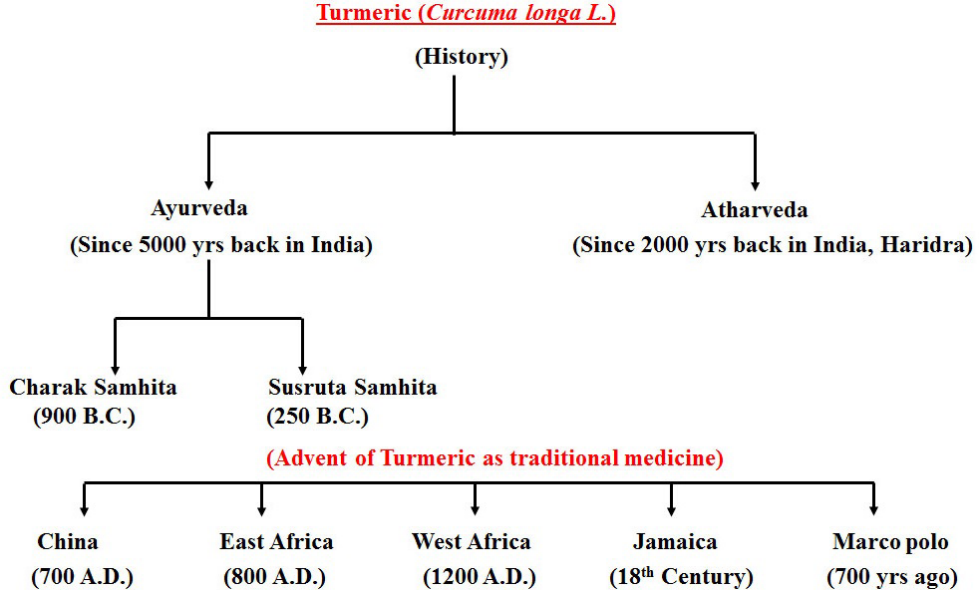
Historical background of Curcumin

**Figure 2:**
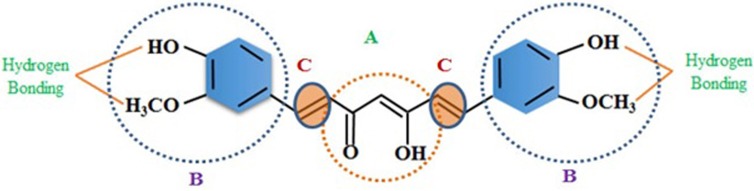
Structure of Curcumin (**A**) b-diketone or keto-enol; (**B**) phenolic; (**C**) alkene linker. Curcumin contains three chemical entities in its structure: two aromatic ring systems containing o-methoxy phenolic groups, connected by a seven carbon linker consisting of an α, β-unsaturated β-diketone moiety.

**Figure 3:**
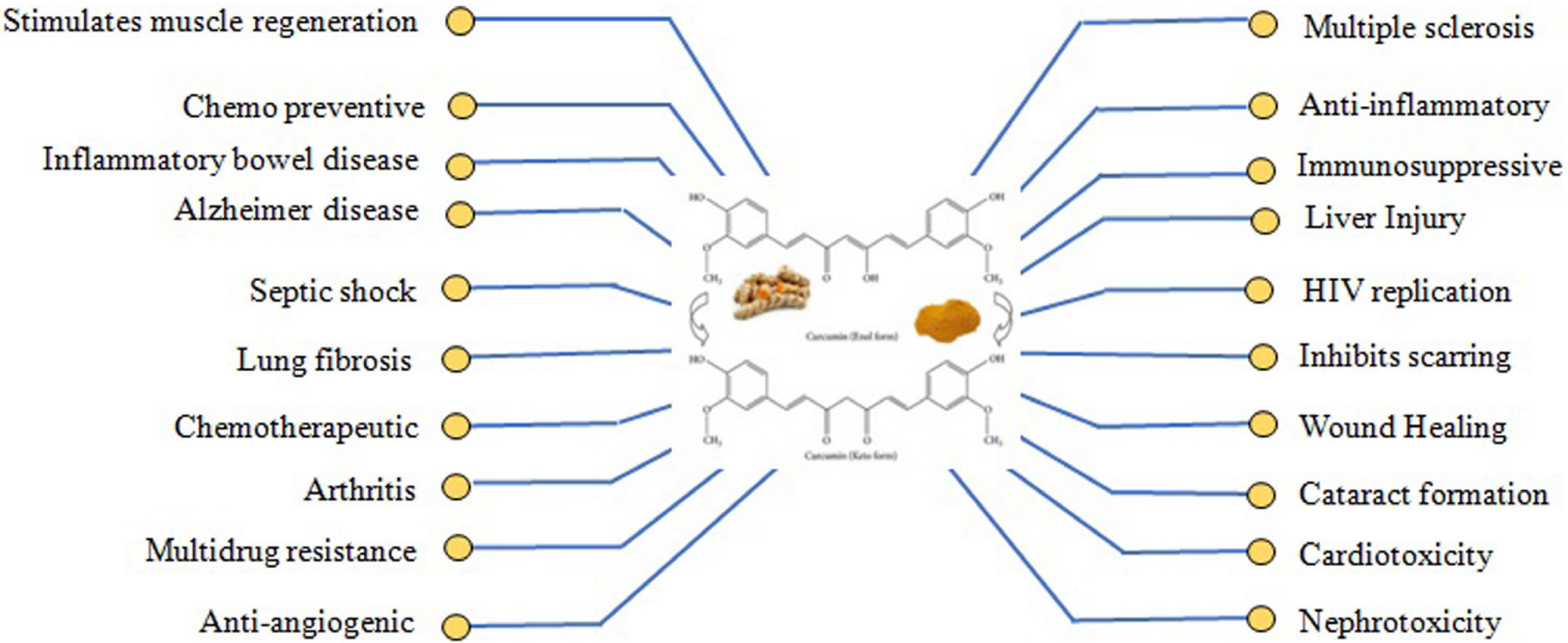
Therapeutic actions of curcumin The Figure illustrates the different pharmacologic effects and therapeutic targets of curcumin.

Since archaic times, people around the world have been harnessing the natural sources for various medicinal purposes. With the passage of time, the curiosity to explore the medicinal benefits of natural habitat is being increased and has now become one of the prime areas of scientific research [1, 2]. *Curcuma longa* (*Linn.*) commonly kenned as turmeric, belongs to Zingiberaceae family and is widely utilized as an ingredient spice [3, 4]. The history of *Curcuma longa* (Linn.) dates back over antediluvian time of Ayurveda, commonly found in tropical, sub-tropical and Southeast regions are widely cultivated areas for use as an ingredient spice (Figure 1). Among a large number of components isolated from turmeric, Curcumin was found to be the most active polyphenol extracted and evidenced by enormous citations in the literature so far [5]. The structure of curcumin (C_21_H_20_O_6_) is also known as diferuloylmethane and first identified by Lampe and Milobedeska in 1910 (Figure 2) [6]. The IUPAC nomenclature of curcumin is 1, 7-bis (4-hydroxy-3-methoxy phenyl)-1, 6-heptadiene-3, 5-dione (1E-6E) consisting of two aryl rings containing ortho-methoxy phenolic OH^–^ groups are symmetrically linked to a β-diketone moiety. The unique polyphenol compound comprises of heptadiene-dione moiety was observed in curcumin with a molecular mass of 368.37g/mole and melting temperature of 183^°^C [7]. Curcumin contains two para hydroxyl groups, keto groups, methoxy groups, an active methylene group [8]. However the solution of curcumin have enol group while it is more stable in keto form [9]. Curcumin is a hydrophobic, polyphenolic compound, hence insoluble in water at acidic and neutral pH conditions, however soluble in methanol, ethanol, dimethylsulfoxide, and acetone [10] with a melting point at 176^°^C ±2. Curcumin exhibits widespread applications such as antioxidant [11, 12], anti-cancer [13], anti-arthritic, anti-microbial [14] anti-diabetic [15] and anti-inflammatory activities [16] and avails in the treatment of many ailments including tendinitis, liver cirrhosis, Alzheimer’s disease, heart attack, hypoglycemia, gastrointestinal problems, worms, swelling, cancer, skin and ocular perceiver infections (Figure 3) [17, 18]. However, the native curcumin is associated with some major drawbacks such as poor absorption, low bioavailability, high metabolic rates and rapid excretion from the body. Hence, in spite of having multidisciplinary medicinal benefits, turmeric has not yet been considered commercially as a potent therapeutic agent.

Despite a century of perpetual research innovations and efforts have aimed to surmount the obstacles of native curcumin, the incursion of nanoparticle formulations may act as a “magic bullet” which rationally incorporated a paradigmatic shift for treating a wide range of chronic diseases utilizing nano-curcumin through efficacious drug distribution process. The numerous studies address the development and *in vitro* evaluation of nano-curcumin that is intended for site-specific delivery of curcumin with high permeability, longer circulation and increased biodistribution which brings significant efficacious responses [19]. Therefore, to enhance the same, various nanoparticle-based approaches have also been sought, such as encapsulation in liposomes [20], chitosan [21], and solid-lipid microparticles based technique utilizing bovine serum albumin [22] to negotiate the various impediments such as poor absorption rate, low bio-availability, and distribution, targeted delivery to the affected tissue of interest which limits its felicitous therapeutic translation. Patra et al., designed a dual (magnetic and thermal)-responsive nanoparticles using advanced nano innovative applications for effective delivery and enhanced efficacy of curcumin [23]. The intranasal administration also identified as a novel nanotechnology based delivery brings about enhancing the bioavailability of curcumin [24].

This observation has led to the lower absorption and poor bio-distribution of native curcuminof medicine holds considerable promise and can resolve these major challenges. To explore its therapeutic potential and to get a molecular insight of the same, nearly 3000 preclinical studies have been conducted that make the consideration of curcumin and its derivatives as one of the most thoroughly investigated and developed natural remedy [25]. This review summarizes the recent investigations on the potential efficacy of curcumin, various nanotechnology-based tools applied for overcoming the limitations and their marked amendments in its restorative utilizations such as anti-oxidant, anti-inflammatory properties, and more concretely anti-cancerous properties. In addition to this, substantial challenges associated with native curcumin which obstructs its clinical translation are additionally discussed.

### Challenges associated with native curcumin as therapeutic agent

The native curcumin has proven to offer enormous therapeutic applications including anti-oxidant [11, 12], anti-carcinogenic [13], anti-microbial [14], anti-diabetic [15] and anti-inflammatory [16], but the utility of curcumin is greatly hindered due to some of its major associated challenges like poor absorption, low bioavailability, rapid systemic elimination and high metabolism inhibiting their therapeutic action. It is noteworthy that curcumin has poor aqueous solubility of about 11 ng/ml [26] and undergoes degradation in alkaline conditions. At pH< 7, the degradation of curcumin is much slower, with less than 20% of total curcumin decomposed at 1 hr [27, 28]. To overcome these major issues, a substantial amount of dosage need to be administered, which reduces its supplemental utilization [29, 30].

Shehzad and his coworkers studied that the oral administration of native curcumin leads to 40% of excretion in the faeces. In addition, in aqueous solution and at neutral pH, curcumin undergoes degradation which might contribute to its short half-life, which is reported to be 28.1 ± 5.6 and 44 ± 7.5 h in the rat for an intravenous dose of 10 mg/kg and an oral dose of 500 mg/kg, respectively [31]. Wahlstrom and Blennow gave the first report by examining the bio-distribution, cellular uptake and excretion of curcumin in Sprague-Dawley rats. Their results concluded that after oral administration of native curcumin at very high amount of dose of about 1g/kg in rats, 75% of which was excreted out in the faeces. They also suggested that only meager amounts of native curcumin were found in the blood plasma levels of rats showed that the native curcumin was poorly absorbed from the gut [32]. The plausible reason of low bioavailability may also be the high metabolic rates thereby reducing bio-distribution as it was also reported that Liver is the major organ involved in the metabolism of curcumin, which undergoes conjugations like glucuronides of Tetrahydrocurcumin (THC) and Hexahydrocurcumin (HHC) in rats [33]. It was also reported that a very small portion of curcumin has been absorbed in intestine which suffered a rapid metabolism in the liver and plasma. Curcumin is extensively converted to its water-soluble metabolites (glucuronides and sulfates) and excreted through urine [34–35]. The administration of radiolabelled [^3^H] curcumin was also studied by Ravindranath et al. in rat intestines at 50–750 µg in 10 ml physiological medium, 30–80% of labeled curcumin was detected in the feces within 72 hrs of ingestion [36]. Moreover, Pan et al. investigated the pharmacokinetic properties of native curcumin using a mouse model, a dosage of 100 mg/kg introduced via. intraperitoneal route and examined that after 1 hour of drug administration, maximum amount was found in the intestine (117 µg/g) while trace amounts were found in other organs like liver, spleen, kidney and brain of about 26.1, 26.9, 7.5 and 0.4 µg/g respectively. This observation has led to the lower absorption and poor bio-distribution of native curcumin [37].

Native curcumin also found to have a surprising potential *in vitro* but exhibits very low or no significant activity *in vivo* due to their poor lipid solubility of 0.6 µg/ml. The nonspecific distribution and inadequate accumulation upon intravenous administration which limits its efficacious response or therapeutic outcome [38]. To achieve the desired therapeutic effectiveness of this natural remedy, curcumin must deliver its active component to the specific site of injury at an optimal rate and amount. The amount of drug and dosage may vary from rapid and consummate absorption when an expeditious onset of action is required during acute conditions such as asthma, heart attack, to slow and sustained in case of longer circulation is needed depending upon their respective therapeutic objective. Thus, further research on curcumin is required to found some possible ways to overcome these limitations. By far, a number of biochemical advancements such as adjuvant merging with other dietary factors, hybridization with metals, liposomal curcumin, phospholipid complexes, conjugated with various polymeric materials and synthetic analogs have been proved to be a promising candidate to improve its bioavailability [39]. However, all of these studies reported the utilization of curcumin in their native form [40].

In addition to the substantial challenges presented individually, the important fact is still need to be noted that these all may vary in their complexity depending on different conditions, such as route of administration (oral vs. intravenous or intraperitoneal), type of disorder encountered (cancer vs. infection) and also the state of disease progression (early vs. late stage cancers) (Table 1).

**Table 1:** Sundry clinical tribulations utilizing curcumin with expected outcomes

Type of Cancer	Effective drug concentration in clinical trials	Route of administration	Outcome	References
**Cervical**	500–12000 mg/day (11 patients)	Oral dose	Curcumin found to be safe and efficacious with no adverse side effects	Cheng et al*.*, 2001 [41]
**Colon**	4 g/day (44 patients)	Oral	Significant reduction in ACF number with five-fold increase in bioavailability	Carroll et al., 2004 [42]
**Head and Neck**	Curcumin tablets 1,000 mg (34 patients)	Orally chewed	Curcumin inhibited IKKß kinase activity in saliva of HNSCC patients with reduced expression of no. of cytokines	Kim et al., 2011 [43]
**Pancreatic**	Gemcitabine+Curcumin (8 gm) (17 patients)	Oral	Extremely safe and feasible for pancreatic cancer patients	Epelbaum et al., 2010 [44]
**Breast**	Curcumin+docetaxel (6 gm/day) (14 patients)	Oral	No adverse side effects were obtained	Bayet-Robert et al., 2010 [45]
**Chronic myeloid leukemia**	Curcumin + imatinib (5 g) (25 patients)	Oral	Showed better efficacy with decreased nitric oxide levels	Ghalaut et al., 2012 [46]

Recently nano-therapeutics showed consequential amendment in therapeutic efficacy by designing and developing its nanoformulations (nanoparticles, nano gels, nanocrystals, liposomes etc.). The bioavailability and efficient delivery of curcumin can be made possible in the form of nano-curcumin which preserves the properties of curcumin and ascertains that it reaches the affected tissue [47]. It is also revealed that the nanoparticles with size ranging from 50–100 nm with marginal negative and positive surface charges are facilely internalized into cancer cells with high bio-interactions between cancer cells and drug particles due to their high contact surface area.

### Role of physicochemical properties of curcumin nanoparticles influencing its therapeutic efficacy

#### Particle size and its distribution

Particle size plays a major role in the mode of action of drug including the interaction of particles with a biological system, tissue distribution, attachment, and rolling [48], firm adhesion of nanoparticles [49], phagocytosis [50], and accumulation [51] are all affected by the size of the particle. Due to the tailoring of a particle in a precise dimension for the purpose of getting the higher rate of absorption and permeation, it ultimately led to an incrementation in bio-distribution and longer circulation *in vivo.* The rate of excretion from the body is also high for large particles (> 1µm), and gets aggregated under physiological conditions and not get filtered from capillaries [52]. In 2011, Liu et al. reported that the curcumin nanoemulsions inhibits 85% of TPA-induced mouse inflammation and also suppresses the expression of cyclin D. This nanoformulation of curcumin showed 3 fold increase in oral bioavailability [53].

Setthacheewakul et al. revealed the self-emulsifying liquid formulations of curcumin are more stable with a particle size of about 30 nm showed 10–14 folds higher rate of absorption compared to the same oral dose of native curcumin (50 mg/kg) administered in Wistar-strain rats [54].

### Surface properties of nanoparticles

The most important characteristics of surface properties of nanoparticles are its surface area, charge present on it and its hydrophilicity. The nano formulation of any particle ranges from 1 nm to 1000 nm, reduction in the size of the material results in an exponential increase in surface area to volume ratio. This may result in enhanced reactivity which dictates its extent of bio-distribution among the tissues and body organs with the rate of excretion from the body.

The surface charge of the nanoparticle is also a paramount feature represents the perpetuated circulation of the drug with enhancement in the accumulation rate at the site of interest. The negatively charged particles have reduced adsorption rate of serum proteins, resulting in longer circulation half-lives [55] as compared to the positively charged particles. The positively charged particles have a high non-specific rate of cellular uptake in the majority of cells.

### Shape of particle

Particle shape is another essential property of nanoparticles that plays a pivotal role in various biological processes associated with its therapeutic activity [56]. The shape of nanoparticle affects its blood circulation, ability to transport, binding affinity, targeting, and internalization into the affected cells and tissues [57]. Geng et al. investigated that the shape of the particle also affects the circulation period of the drug, they observed the polymer micelles (filo micelles) of filamentous in shape have long-circulating lifetimes (>1 week after administration) compared with spherical counterparts (2–3 days) [58]. The shape of nanoparticles contributes to their interaction with cell membranes. Recent studies have indicated that the oblate shape of particles favors their circulation in the blood due to lower uptake by macrophages [59]. Subsequently, it also increases their blood circulation period and targeted delivery to the site. The shape of nanoparticle also effected its bio distribution and macrophage uptake level. For a nanoparticle, to increase their circulation time and retention power in the cell, it must first be able to travel in the bloodstream while evading uptake by macrophages, particularly in the reticuloendothelial system. Biodistribution studies have also demonstrated that the uptake of spherical particles is favored over the uptake of particles with high aspect ratios in macrophages. The tailoring of nanoparticle shape and dimenison also has improved the efficacy of tumor therapy. For example, trastuzumab coated nanorods exhibited a 5-fold greater cellular growth inhibition of when BT-474 breast cancer cells compared to equivalent nanospheres at the same nanoparticle dose [60]. This leads to increase of upto 66% in binding ability and uptake by the cells of the nanorods compared to the nanospheres.

### Effect of aggregation and agglomeration on particle

The aggregation and agglomeration of nanoparticles is seems to be a ubiquitous phenomenon which affects in mediating cellular uptake and interactions. The formation of large NP complexes in suspension is frequently referred to as ‘aggregation’ or ‘agglomeration’. It occurs due to the effect of van der Waals attractive forces between particles are greater than the electrostatic repulsive forces produced by the nanostructure surface [61, 62]. They both effects dramatically with many aspects of nanoparticle-cell interactions. Particle agglomeration is influenced by a number of factors, including primary particle characteristics as well as the properties of the medium that the particles are suspended in [63]. The aggregation states of nanoparticles also influence their toxicities. Fundamentally, the aggregation states of nanoparticles depends on size, surface charge, and composition among others. It has been observed that carbon nanotubes are mainly accumulated in liver, spleen, and lungs without manifesting any acute toxicity but induce cytotoxic effects mostly because of accumulation of aggregates for longer periods [64]. Agglomerated carbon nanotubes have more unpropitious effects than well-dispersed carbon nanotubes and enhance the pulmonary interstitial fibrosis [65].

### Encapsulation efficiency of particle* *

The rational design strategy of encapsulation efficiency or surface coating of nanoparticle greatly influences its solubility and interaction with the other biological entities. It also affects their chemical reactivity and pharmacokinetic property of nanoparticle [66]. The nanoparticle encapsulation efficiently overcomes many of its impediments like targeted drug delivery approach, cellular uptake, biodistribution and accumulation at the site of interest.

Many researchers used polymeric materials like Poly (lactic-co-glycolic) acid (PLGA), Polyethylene glycol (PEG), surfactant copolymers for encapsulating the nanoparticles with the purpose of maintaining the higher stability of the nanoparticulate system in biological milieu [67]. Recently, the neuronal uptake and neuroprotective effects of curcumin loaded PLGA nanoparticles in the human SK-N-SH cell line have been observed [68]. It has also been shown that curcumin encapsulated in PLGA nanoparticles may induce neurogenesis and reverse cognitive deficits in Alzheimer’s disease model [69]. Moreover, many polymeric nanoparticles composed of N-isopropylacrylamide (NIPAAM), vinylpyrrolidone (VP), and acrylic acid (AA) containing curcumin (NanoCurc™) protected human SK-N-SH cells from ROS hydrogen peroxide (H_2_O_2_)-mediated cell damage [70, 71] Mulik *et al* showed that apolipoprotein E3 (ApoE3) mediated PnBCA nanoparticles containing CUR exhibit neuroprotective action against A*β*-induced cytotoxicity in SH-SY5Y cells [72, 73].

### Drug content and release profile of nanoparticle system

The content of drug loaded and its release profile at the site of interest of any nanoparticulate system is highly dependent on the type of nanoparticle used and its method of preparation. In the case of curcumin nanoparticles, the amount of drug encapsulated has been quantified using different estimation methods [74–76].

Zhu et al. studied the drug release profile of curcumin encapsulated with poly (butyl cyanoacrylate) nanoparticles and observed 34.7% release in 2 hours followed by a sustained release using standard two-phase kinetics equation: 100 − Q = 4.5235e (−0.1724t) + 4.1641e(−0.0114t) [77]. In most of the nanoformulations of curcumin, the *in vitro* release kinetic follows a biphasic pattern. The sustained release profile of curcumin nanoparticles may vary depending on different factors such as nano formulation, its composition, the location of entrapment and amount. The rational tailoring of nanoparticle design and prepared in such a way with an intrinsical capacity of high drug entrapment efficiency and loading capacity. The drug loaded and release is an important factor as the therapeutic potential of curcumin nanoformulation is greatly dependent on its release in its active form at the targeted site of interest.

### Nanotechnological interventions of native curcumin to overcome its inherent constraints

The emergence of nanotechnology has now caused the convergence of experimental advancements for the welfare of society with a wide range of applications and use of nano-scale phenomena with novel properties for a felicitous remedial action. Nanotechnology has proved to be a very efficacious implement for various strategies accentuated ecumenically to escalate the constraints of native curcumin for the enhancement of its therapeutic potential coupled with some important factors, including high cellular uptake, biodistribution, dissolution rates, good blood stability and controlled drug release at the site of injury.

**Figure 4:**
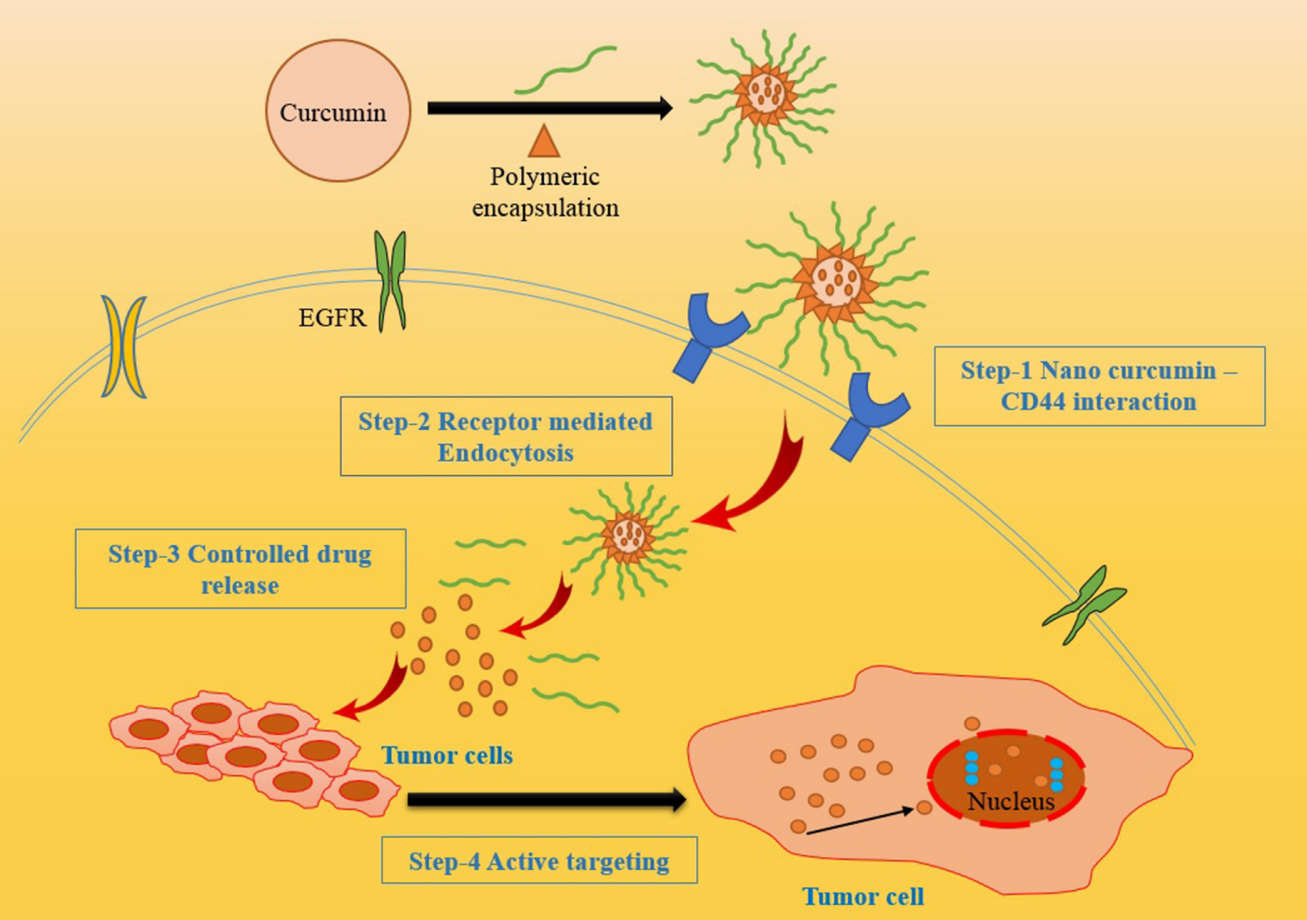
Different nano carriers of curcumin for enhanced drug delivery system The various nano-carriers with effective encapsulation strategies to enhance the targeted drug delivery.

The different types of nano-carriers were utilized for curcumin nanoparticle formulation such as polymeric nanoparticles, solid-lipid nanoparticles, polymeric micelles, curcumin nanocrystals, nano-emulsions, nano liposome-encapsulated curcumin, Cyclodextrin, curcumin nanosuspension and dendrimers. They provide protracted blood circulation, better permeability, and resistance to metabolic processes [78] [Figure 4]. A number of studies were carried out to elucidate the different nano formulations of curcumin for effective therapeutic activity (Table 2).

**Table 2:** Nano-technological alteration in herbal drug curcumin along with their expected benefits

Nanotechnology tool	Size and shape	Models used	Outcome	References
Curcumin encapsulated with liposomal PMSA antibodies	100–150 nm	Human Prostate cancer cell lines (LNCaP and C4-2B	Showed better efficacy(70–80% inhibition in cell proliferation) with enhanced targeted delivery	Thangapazham et al., 2008 [79]
Curcumin/MPEG-PCL micelles	27.3 ± 1.3 nm	Colon carcinoma cell (C-26)	Excellent inhibition of cancer growth by inhibiting angiogensis	Gou et al., 2010 [80]
Curcumin loaded PBCN nanoparticles	152.0 nm	Alzheimer’s disease	Showed excellent transport of curcumin to brain	Sun et al., 2010 [81]
Curcumin nanoemulsion	< 200 nm	human ovarian adenocarcinoma cells (SKV3) and drug resistant (SKOV-3TR )	Showed significantly increased cytotoxic activity	Ganta et al., 2009 [82]
Curcumin-PLGA nanoparticles	248 ± 1.6 nm	Erythroleukemia type K562 cells	Improved clinical management of leukemia	Misra et al., 2011 [83]
Curcumin-Chitosan nanopartilces	100–250nm	Melanoma tumors	Increased antitumor and anti-cancer effects	Li et al., 2012 [84]
Nanogels (cross linked polymer network)	10–200 nm	Breast and pancreatic cancer cells	Increased stability with enhanced anticancer effects	Mangalathillam et al.2012 [85]
Curcumin-nanocrystal solid-dispersion	250 nm	Pharmacokinetic properties	Improved physiochemical andpharmacokinetic properties.	Onoue et al., 2010 [86]

Recently, Sasaki et al. reported curcumin encapsulated into methoxy poly (ethylene glycol) poly (Ɛ-caprolactone) nanoparticles with effective drug delivery and enhanced oral bioavailability [87]. The encapsulation of curcumin with more than 97.5% potency in PLGA and PEG was studied by Garodia et al. and proved that the encapsulation shows higher efficacy and faster cellular uptake than the native curcumin *in vitro* [88]*.*

Jourghanian et al. synthesized curcumin loaded solid-lipid nanoparticles by high-pressure homogenization method using mannitol as cryoprotectant and cholesterol as a carrier which enhances its stability and biocompatibility [89]. Another study demonstrated a novel folate-conjugated, curcumin-loaded human serum albumin nanoparticles (F-CM-HSANPs) prepared by the chemical conjugation of folate to the surface of curcumin loaded human serum albumin nanoparticles injected *in vitro* results in sustained drug release at desired site and prolonged retention time with specific targeting *in vivo* after the intravenous injection of F-CM-HSANPs in current clinical tribulations [90].

The cyclodextrin-based nanosponges of curcumin cross-linking with dimethyl carbonate were synthesized and formulated the intricate of ß-cyclodextrin-curcumin nanosponge by Darandale et al. which significantly enhanced the stability as well as solubility compared to free curcumin. Also, the *in vitro* drug release efficacy of curcumin was found to be highly controlled over a prolonged duration and found to be non-hemolytic [91].

Sun *et al.,* developed a cationic liposome containing PEI-PEG as a carrier involute (LPPC) encapsulate curcumin with enhanced anti-tumor effects on colon/melanoma tumor growth in mice. It was found that curcumin/LPPC intricate exhibits enhanced the anti-proliferative effect and is able to rapidly perforate into the cells [81].

In another study conducted by Mangalathillam et al., reported curcumin loaded chitin nano gels comprised of cross-linked polymer network tested *in vitro* on breast cancer cell lines and observed an amelioration in bioavailability, anticancer effects, better-controlled release and enhanced stability [85].

Kundu et al. reported curcumin-loaded lipid nanoparticles and investigated anti-glioma activity in encephalon tissue for effective glioblastoma therapy resulting in enhanced bioavailability. They demonstrated that curcumin-loaded nanoparticles inhibited cellular proliferation, migration, and incursion along with a higher percentage of cell cycle arrest and telomerase inhibition, thus leading to a more preponderant percentage of apoptotic cell death in glioma cells compared with native curcumin. Thus, curcumin-loaded nanoparticles can be utilized as an effective delivery system to surmount the challenges of drug delivery to the brain, providing an incipient approach to glioblastoma therapy [92]. These novel strategies significantly ameliorated curcumin’s aqueous solubility, cellular uptake, controlled release with enhanced dissolution rates.

**Figure 5:**
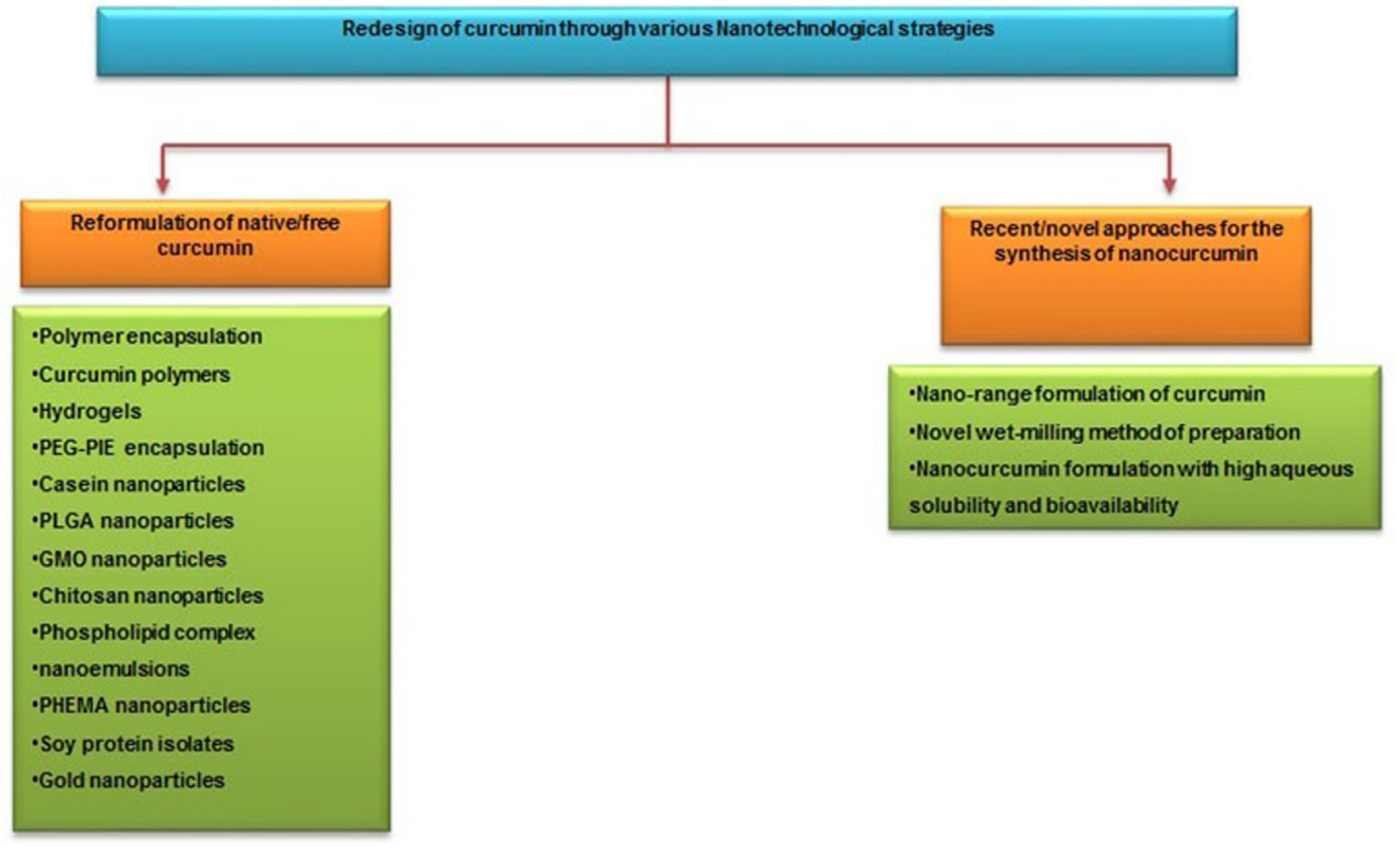
Comparative study of different strategies used for the preparation of nano curcumin for amended drug uptake and bio distribution

Recently, researchers from Johns Hopkins University School of Medicine and the University of Delhi have jointly developed a polymer nanoparticle-encapsulated form of curcumin, “nano-curcumin”, which can be readily dispersed in aqueous media. In this process, they have coated ordinary hydrophobic curcumin particles with a hydrophilic polymer (N-isopropyl acrylamide) with N-vinyl-2-pyrrolidonne and poly (ethylene glycol) monoacrylate nanoparticles. This nano formulation increases aqueous solubility and can be readily absorbed into the blood stream. It has already been tested *in vitro* on pancreatic cancer cells and it was shown to have equal or better effects than free curcumin on the human cancer cells, such as inhibition of NF-kB and down-regulation of interleukin-6 (IL-6) [93]. The comparative study of various nanotechnological strategies used for the preparation of nanoformulation of native curcumin using different encapsulation methods and the novel approach of converting native curcumin absolutely in a nano range without any encapsulation are illustrated in Figure 5.

### How curcumin nanoformulations affects its pharmacological behavior?

**Figure 6:**
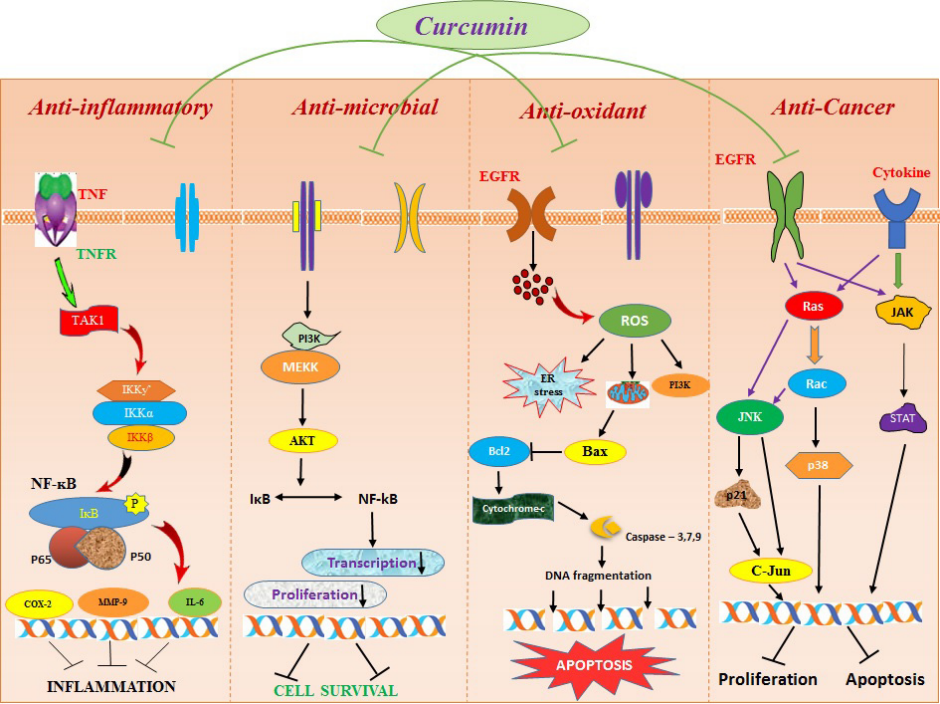
Mechanism of action of curcumin in different biomedical applications The Figure illustrates the detailed mode of action of curcumin mediated for different therapeutic applications by entering inside the cell through appropriate cell surface receptors with efficacious targeted distribution and enhanced response by regulating various cytokines and induces apoptosis in cancer cells.

Nanoformulations of curcumin greatly transmuted the way of treating diseases by amending its bio-availability, cellular uptake, and permeability with enhanced plasma concentration. Curcumin nanoparticles efficaciously distribute the exact therapeutic concentration of drug at the site of injury. Numerous studies have been reported on the therapeutic efficacy of curcumin nanoformulations and explored its consequential properties against the wide range of human diseases [94]. The pharmacophore elements in curcumin such as central 5-hydroxy-1, 7-diarylhepta-1, 4, 6-trien-3-one and 3-methoxy-4-hydroxyl aryl rings and α, β unsaturated carbonyl, confer a large variety of biological activities, involved in cellular defense against oxidative stress and chemo-preventive effect [95] and its detailed mechanism of action has been illustrated in Figure 6. Recent research and numerous studies on animals have also focused on the adequacy and safety in the utilization of curcumin nanomedicine on several types of biological activities. [96].

### Anti-inflammatory activity of curcumin nanoformulations

The nano formulations of curcumin have potentially amended the major obstacles of native curcumin such as low solubility, instability, poor bioavailability and rapid metabolism. Consequently, curcumin nanoparticles gained immense attention to improve its profound activity against diverse inflammatory conditions such as bronchial asthma, uveitis, periodontitis, inflammatory bowel diseases etc [97]. In 2015, Abdul Mohsen et al. prepared amorphous nano-curcumin using water titration method and evaluated its anti-inflammatory effects *in vitro* and *ex vivo* in carrageenan-induced paw edema method in rats with Diclofenac as standard and concluded that NanoCur was highly significant and efficacious as compared to native curcumin [98].

The anti-inflammatory activity of curcumin complexes is reported due to the presence of 4-hydroxyphenyl unit which is further increased by the integration of acylation and alkylation or methoxy groups on the phenyl ring of curcumin [99]. As an anti-inflammatory, curcumin causes downregulation of NF-kB, cyclooxygenase 2 (COX2) and pro-inflammatory cytokines such as interleukin-1 (IL-1 and IL-6) and TNF-α and showed high efficacy against rheumatoid arthritis, psoriasis, and post-operative inflammation [100]. Curcuminoid showed marked improvement in patients and was also found to be safe and highly efficacious throughout the clinical trial [101, 102].

Oppenheimer et al, conducted the first clinical tribulation of native curcumin in human biliary diseases to investigate the effect of “curcumen” by intravenous injection of 5% sodium curcumin solution which showed rapid vacating of the gallbladder and patient showed consummate remedy [103].

Another study was conducted among 16 patients suffering from chronic kidney disease to evaluate the anti-inflammatory effect of a curcuminoid and found to be mediated by regulating the inflammatory mediators such as IL-6, IL-8, TNF-α, TNF-ß, substance P, hs-CRP, CGRP, and MCP-1. Curcuminoid showed marked amelioration in patients and was additionally found to be safe and highly efficacious throughout the clinical trial [104]. Another study on rat intestine showed that curcumin enhanced the expression of SOCS-1, via down-regulation of JAK2/STAT3 signaling [105]. The anti-inflammatory effect of Cyclodextrin-mediated curcumin complex was evaluated and found that Cyclodextrin-curcumin intricate showed higher affinity than native curcumin in inhibiting the inflammatory transcription factor, such as nuclear factor kappa-b (NF-κB) and also tested for the treatment of inflammatory bowel disease in rat model. [106, 107].

### Anti-oxidant activity of nano-curcumin

The structure of curcumin contains a carbonyl, methoxy and hydroxyl groups pertaining to its antioxidant activity due to its ability to scavenge free radicals *in vivo*, especially peroxyl radicals (ROO^.^). Albeit curcumin contains a β-diketone moiety which may subsist in a cis, Trans and enol form. Tonnesen et al reported that curcumin subsits in the cis-enol form in solutions [108].

Vajragupta et al. demonstrated inhibition of oxidative stress by curcumin in humans during exercise and also found decremented astringency of pre-menstrual syndrome in women by regulating the neurotransmitters and biomolecule levels. The defensive properties of curcumin are attributed a symmetrical di phenolic dienone series which includes members that retained or were devoid of phenolic groups [109].

Studies revealed that expression of 2 and 4- hydroxyphenyl units and an ortho alkoxy group contributed to the enhancement of antioxidant activity [110, 111]. However, under the situations of extortionate engenderment of peroxynitrite and hydrogen peroxide oxidants, curcumin react with peroxyl radicals and composed phenoxyl radicals which show less reactivity than the peroxyl radicals. Further, it was reported that polyethylene glycosylated (PEGylated) curcumin analogs for potent nuclear factor erythroid-2 cognate factor 2 (Nrf2) activators that regulate the antioxidant defense system and act as modifiers for inflammatory diseases [112]. Few studies have revealed up-regulation of endogenous cellular antioxidant contrivances following curcumin administration that contributed the initiation of cytoprotective Nrf2-induced target genes which scavenged reactive oxygen species (ROS) thereby protecting cells from ROS-induced oxidative stress [113–115].

In recent years an incipient biopolymer micelle of curcumin has been prepared by encapsulating with modified Ɛ-polylysine (M-EPL) micelle and demonstrated that M-EPL encapsulation effectively stabilized curcuminoid showed elevated cellular antioxidant activity compared with native curcumin [116].

### Anti-microbial activity of nano-formulated curcumin

The development of resistance against subsisting microbial drugs has become a critical quandary for therapeutic strategies. Due to the antimicrobial activities of native curcumin, the research has been expedited to get insight into the aspect of controlling pathogens. This polyphenol compound has shown a wide range of activity against various microorganisms such as *Bacillus subtilis*, *Staphylococcus aureus*, *Pseudomonas aeruginosa*, *Escherichia coli*, *Aspergillus niger*, *Penicillium notatum*, *Salmonella paratyphi, Mycobacterium tuberculosis* and certain pathogenic fungi [117, 118].

Nanoformulation of curcumin possesses enhanced antibacterial potential activity than native curcumin which is attributed to its improved aqueous-phase solubility and dispersing ability. Curcumin induced membrane permeabilization is involved in disordering the 1, 2-dipalmitoyl-sn-glycero-3-phosphocholine (DPPC) membranes [119].

Curcumin inhibits the FtsZ polymerization thereby suppress the FtsZ assembly possibly leading to disruption of *E. coli* and *B. subtilis* proliferation [120]. In 2015, Gera et al. fabricated a nano-curcumin loaded medicated patch with controlled drug release efficacy and revealed that the nano-curcumin coated bandage showed enhanced bioactivity against different pathogenic and non-pathogenic microbial strains [121]. The encapsulation of curcumin with chitosan-PVA-silver nanocomposite films has been studied by Vimala et al. as an anti-microbial wound/burn dressings and identified enormous growth inhibition of *E. coli* in comparison with native curcumin or chitosan-PVA-silver nanoparticles film discretely [122].

Curcumin also showed significant therapeutic potential against *Helicobacter pylori*. An explanatory study in *Helicobacter pylori*-infected C57BL/6 mice utilizing curcumin exhibited eradication effect against the infection and restored gastric damage [14]. The anti-fungal activity of curcumin against pathogens derived from food such as *Penicillium notatum, Aspergillus niger* and *Saccharomyces cerevisiae* has shown the prominent possibility for the utilization in the pabulum industry [123]. The potential mechanism of antifungal activity of curcumin is due to the breach in the integrity of plasma membrane which caused leakage of potassium ion from the cytosol and transmute in membrane potential leading to cell death. The study also suggested that downregulation of desaturase (such as ERG3) leading to significant reduction in ergosterol of fungal cell lead to cell death via generation of ROS [124].

The antiviral activity of curcumin has been reported against numerous viruses, including influenza virus infection (IAV), human papilloma virus (HPV), coxsackievirus, Hepatitis C virus (HCV), adenovirus and Herpes simplex 1 (HSV-1). It is well documented that curcumin is a strong inhibitor of NF-kB signaling that might impact upon IAV propagation [125]. *In vitro* study using curcumin and its derivatives exhibited remarkable antiviral activity against herpes simplex virus type 1 (HSV-1) [126]. The coalescence of curcumin and IFNα inhibited the HCV viral replication through the Akt-SREBP-1 activation thereby inhibiting HCV gene expression [38]. Undeniably, adscititious studies are still needed to attest the usefulness of curcumin in the treatment of patients with highly prevalent viral infections. Apart from some reports on the therapeutic efficacies of curcumin, research areas nowadays are majorly focused on the utilization of nano-curcumin for its immune-modulatory and antimicrobial effects against HIV and other pernicious microbes such as infiuenza virus, adenovirus, cox sackie virus and Mycobacterium tuberculosis.

### Anti-cancer activity of nanoformulation mediated curcumin

The influx of nanotechnology brings an abundance of opportunities for elongating the therapeutic activities of this natural remedy, curcumin by improving its aqueous dispersion, bioavailability and cellular uptake. Curcumin is a highly pleiotropic molecule which shows positive efficacy as a chemopreventive agent against the malignant tumor proliferation in colon, breast, prostate, esophagus and lung etc.

**Figure 7:**
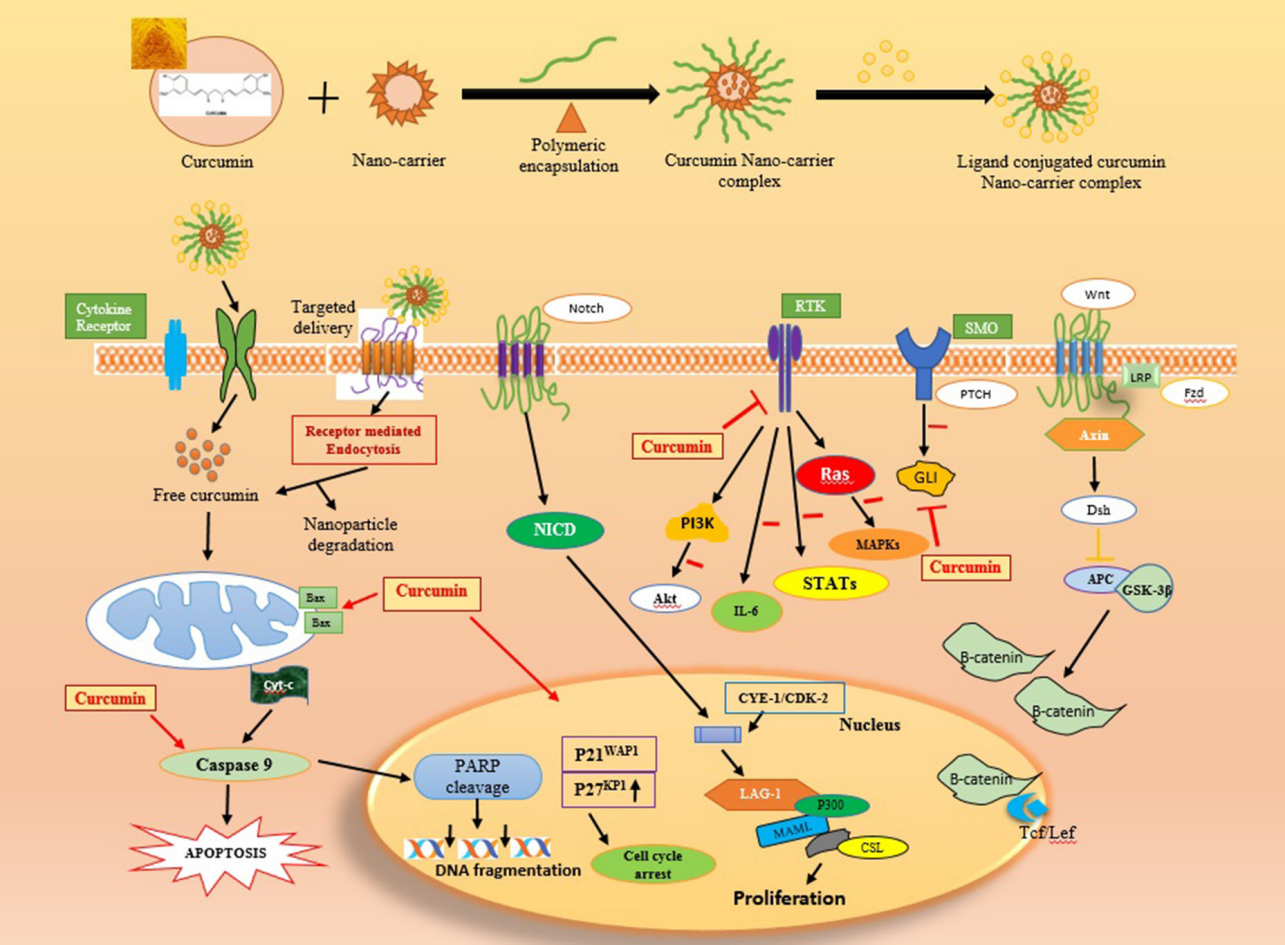
Multiple anti-cancer cascades of curcumin by different growth factors in cancer cells The inhibitory effect of curcumin on the expression and/or activity of EGFR, erbB2, IGF-1R, and their downstream signaling elements, sonic hedgehog (SHH/SMO/GLIs), Wnt/β-catenin and ATP-binding cassette multidrug transporters such as ABCG2 in cancer cells are indicated. Moreover, the enhanced expression of p21^WAP1^ and p27^KIP1^ Cyclin-dependent kinase inhibitors and inhibition of mitotic effects induced by curcumin resulting in a cell cycle arrest and reduced expression levels of different gene products involved in the growth, invasion and metastasis of cancer cells as well as the activation by curcumin of mitochondrial factors and caspase pathway-induced apoptosis are also indicated.

Curcumin has attracted great attention in the therapeutic activity in clinical oncology due to its chemo preventive, antitumor, radio sensibilizing and chemosensibilizing activities against various types of cancer cells. These malignancies include breast, ovarian, prostate, leukemia’s, lymphomas, multiple myeloma, brain cancer, melanoma and skin and lung cancers. Curcumin mediates its anti-proliferative, anti-invasive and apoptotic effects on cancer cells through multiple molecular mechanisms. The oncogenic pathways inhibited by curcumin encompass the members of epidermal growth factor receptors (EGFR), sonic hedgehog (SHH)/GLIs and Wnt/β-catenin and downstream signaling elements such as Akt, nuclear factor-kappa B (NF-κB) and signal transducers and activators of transcription (STATs) (Figure 7) [127]. In counterbalance, the high metabolic instability and poor systemic bioavailability of curcumin limit its therapeutic efficacy in human. Of great therapeutic interest, the selective delivery of synthetic analogs or nanotechnology-based formulations of curcumin to tumors, alone or in combination with other anticancer drugs, may improve their chemo preventive efficacies against cancer progression. Novel curcumin formulations may also be used to inhibit the drug resistance, eradicate the total cancer cell mass and improve the anticarcinogenic efficacy in patients.

Studies have indicated the therapeutic efficacy of curcumin has been considered as safe and potent drug and exhibits no major toxicity and only protects normal cells and organs at least in part by up-regulating the nuclear factor erythroid-derived-2 related factor 2 (Nrf2)-induced antioxidant gene products. In the studies of Zaman et al., 2016 prepared poly (lactic-co-glycolic acid) predicated curcumin nanoparticle formulation and demonstrates that the polymeric nanoparticle efficaciously inhibits the cell growth, induces apoptosis and cell cycle arrest in cervical cancer cell lines as compared to the native curcumin. [128].

Mukherjee and Vishwanatha et al., prepared the nanospheres of curcumin with encapsulation of PLGA and evaluated against prostate cancer cell lines, LNCaP, PC3, and DU145 revealed that the IC_50_ value of cells treated with polymer encapsulated nano-curcumin is less than the native or free curcumin treated cells [129]. Various polymeric nano formulations of curcumin were synthesized by crosslinking of N-isopropyl acrylamide, N-vinyl-2-pyrrolidone, and poly (ethylene glycol) monoacrylate and studied its anti-proliferative activity against pancreatic cancer cells and amended results were obtained in the case of polymer loaded nanoparticles as compared to native curcumin [93].

The anticancer activity was also studied against breast cancer cell lines by induction of apoptosis using Transferrin-mediated solid lipid nanoparticles (SLNs) of curcumin and the flow cytometric results concluded that the transferrin-mediated SLNs nano-curcumin has potentially gives enhanced anticancer activity of curcumin in breast cancer cell lines *in vitro* compared to the native or free curcumin [130].

Extensive studies, both in cancer cell lines and animal tumor models have reported the higher cellular uptake in nano-formulated curcumin in cancer cells than native curcumin by inhibition of chemokine and metastasis, thereby delaying or inhibiting the proliferation [131, 132]. The multiple mechanisms involved in cell proliferation (Cyclin D1, c-Myc), cell survival (Bcl-2, Bcl-xL), and apoptosis (caspase -8, 3, and 9) may mediate the permissive chemotherapy and chemopreventive effects for a selective targeting of highly proliferating cancer cells over normal cells [133, 134]. At the molecular level, the anticancer effects of curcumin are underlying the mechanism of action through inhibiting the expression of the specificity protein (Sp) transcription factors Sp1, Sp3, and Sp4 [135, 136].

The suppression of malignant tumor progression utilizing curcumin revealed the diverse and complex mechanism of action by up-regulation of pro-apoptotic proteins such as Bim, Puma, Bax, Bak, Noxa; downregulation of anti-apoptotic proteins such as XIAP, Bcl-2, and Bcl-xL; growth factor receptors (such as EGFR, HER2) and inhibits the activity of c-Jun N terminal kinase [137], protein tyrosine kinases and protein serine/threonine kinases which reduced the metastatic activity [138].

Curcumin has many molecular targets and has a diverse and complex mechanism of action. Curcumin regulates the transcription factors of NF-ƙB resulting in Upregulation of the expression of anti-apoptotic genes expression that is implicated to be involved in carcinogenesis [139]. The curcuminoid showed a reduction in cancer cell proliferation by suppressing the cell cycle regulatory proteins Cyclin E, Cyclin D1 Cyclin B [140]. The MMPs family member proteins are reported to play a vital role in malignant tumor progression and metastasis [141]. *In vivo* study showed that curcumin inhibits the expression of MMP-2 and MMP-9 in B16F-10 melanoma cells [142].

From last few decades, the investigations have explored the curative effects of curcumin in endometrial and ovarian cancer cells on the negative controllers of activated STAT-3 like SHP-1, SHP-2, SOCS and PIAS [143, 144]. The coalescence effect of curcumin and docetaxel treatment against multidrug resistant tumors in mice ovarian has shown significant reduction about 58% in growth of the tumor. The report demonstrated that administration of curcumin (500 mg/kg/day) leads to increment in apoptotic activity of tumor cells, reductions in the growth of tumor up to 49–55% and microvessel density of normal ovarian tumor cells [145].

The tgf-β signaling pathway is a tumor inhibitor, but alterations in TGF-β signaling pathway promotes cancer. The cytotoxic effect of curcumin on colon and cervical cancer cell lines via inhibiting the TGF-β signaling cascade [146]. It is downregulated TGF-β signaling pathway such as p21, cyclinD1, and Pin1 by lowering the expression of Smad4, P-Smad3, and TGF-β Receptor II. The study in mouse xenograft model showed the chemopreventive effect of curcumin on colorectal cancer cell lines regulates the tumor-suppressive miR-34a, specific miRNAs, and down expression of miR-27a.

Apart from some reports on the therapeutic efficacies of nano-curcumin for the treatment of parasitic diseases, microbial diseases and toxicity, most research areas are majorly focused on the utilization of nano-formulated curcumin for cancer therapy, showed improved targeted anticancer effect compared to native curcumin [147]. In a polar medium, curcumin has the unique ability to perforate into the cell and with an excellent electron transfer capability can facilely enter into the plasma membrane of the cell. Hence, the particle size of curcumin nanoparticles demonstrated better aqueous phase solubility and having much more vigorous anticancer effect against various cancer cell lines [148]. Consequently, nano formulations of curcumin showed ameliorated therapeutic efficacy compared to bulk or native curcumin.

## CONCLUSIONS

Nanotechnology is not only the stream of infinite possibilities but a burgeoning field for prospective future, but it is realistic to many scientists and investigators working to accomplish incredible goals every day. The current review article accentuates on a native medicinal herb “Turmeric” and its active ingredient, *“Curcumin”* as a unique therapeutic agent. Despite its incredible multi-targeting potential *in vitro*, its pharmacological efficacy is obstructed *in vivo* due to some major challenges associated such as poor aqueous solubility, high metabolism, rapid excretion and infelicitous molecular size resulting in poor systemic availability, etc. Consequently, to surmount these major obstacles, the nano-technological intervention plays a consequential role in converting this old-age remedy to an age-old solution with amended curative index. A well-formulated nano approach leads to an enhancement of bioavailability and bioactivity by reducing the particle size, modification of surfaces, and entrapment of curcumin with different nanocarriers. The avenue of nanotechnology could improve the perspective for medical patients with serious illnesses or injuries. Nanotechnology itself revolutionizes many different aspects of our lives and also proven to be a wondrous and majestic engenderment in near future.

## Abbreviations

THC: Tetrahydrocurcumin
HHC: Hexahydro-curcumin
TPA: 12-O-tetradecanoylphorbol-13-acetate
PLGA: Poly (lactic-co-glycolic acid)
PEG: Polyethylene glycol
F-CM-HSANPs: folate-conjugated, curcumin-loaded human serum albumin nanoparticles
DPPC: 1,2 dipalmitoyl-sn-glycero-3-phosphocholine
IL-6: Interleukin-6
NF- kB: Nuclear Factor kappa-b
COX2: Cyclooxygenase 2
TNF-α: Tumor necrosis factor-alpha
CGRP: Calcitonin Gene Related Peptide
MCP-1: Monocyte Chemo attractant Protein-1
SOCS 1: Suppressor of cytokine signaling 1
JAK2/STAT3: Janus kinase 2/ Signal transducer and activator of Transcription 3
ROS: Reactive oxygen species
M-EPL: Modified Ɛ-polylysine
DPPC: dipalmitoyl-sn-glycero-3-phosphocholine
IAV: Influenza avian virus
HPV: Human papilloma virus
HSV-1: Herpes simplex virus type 1
HCV: Hepatitis C virus
MMP-2: Matrix metalloproteinase-2
MMP-9: Matrix metalloproteinase-9
TGF-β: Transforming growth factor-beta
SHP1: Src homology phosphatase-1
PIAS: Protein inhibitor of activated signal (Protein)
XIAP: X-linked inhibitor of apoptosis protein
